# Impaired Brain Information Transmission Efficiency and Flexibility in Parkinson's Disease and Rapid Eye Movement Sleep Behavior Disorder: Evidence from Functional Connectivity and Functional Dynamics

**DOI:** 10.1155/2022/7495371

**Published:** 2022-09-15

**Authors:** Jing Wei, Jiaqi Lyu, Jie Xiang, Yan Niu, Lan Yang, Chanjuan Fan, Dandan Li, Yanli Yang

**Affiliations:** ^1^College of Information and Computer, Taiyuan University of Technology, Taiyuan 030024, China; ^2^School of Information, Shanxi University of Finance and Economics, Taiyuan 030006, China

## Abstract

Parkinson's disease (PD) is a common neurodegenerative disorder. Rapid eye movement sleep behavior disorder (RBD) is one of the prodromal symptoms of PD. Studies have shown that brain information transmission is affected in PD patients. Consequently, we hypothesized that brain information transmission is impaired in RBD and PD. To prove our hypothesis, we performed functional connectivity (FC) and functional dynamics analysis of three aspects—based on the whole brain, within the resting-state network (RSN), and the interaction between RSNs—using normal control (NC) (*n* = 21), RBD (*n* = 24), and PD (*n* = 45) resting-state functional magnetic resonance imaging (rs-fMRI) data sets. Furthermore, we tested the explanatory power of FC and functional dynamics for the clinical features. Our results found that the global functional dynamics and FC of RBD and PD were impaired. Within RSN, the impairment concentrated in the visual network (VIS) and sensorimotor network (SMN), and the impaired degree of SMN in RBD was higher than that in PD. On the interaction between RSNs, RBD showed a widespread decrease, and PD showed a focal decrease which concentrated in SMN and VIS. Finally, we proved FC and functional dynamics were related to clinical features. These differences confirmed that brain information transmission efficiency and flexibility are impaired in RBD and PD, and these impairments are associated with the clinical features of patients.

## 1. Introduction

Parkinson's disease (PD) is a neurodegenerative condition characterized by motor and nonmotor symptoms; cognitive decline and sleep dysfunction are among the most common symptoms [1]. Rapid eye movement sleep behavior disorder (RBD) was identified as a key prodromal symptom of PD by the Movement Disorder Society [2]. It can also occur in the course of PD [3]. The study of RBD is significant for the diagnosis of PD.

Studies have shown that the brain network of RBD and PD is damaged [3, 4]. Human brain functional connectivity (FC) network, estimated based on resting-state functional magnetic resonance imaging (rs-fMRI), has been widely utilized to study brain functional networks in RBD and PD. Several studies have investigated that the global efficiency of FC was decreased in PD patients [5, 6], and these results proved that the brain information transmission efficiency of the whole brain is decreased. Researches have found that PD is accompanied by FC loss of the sensorimotor network (SMN), both within the network and in interaction with other networks [7]. In RBD, only a few FC studies have been reported, focusing on abnormalities in motor-related networks [8]. The abnormality of FC implies the decline of brain information transmission ability. Given the above, some studies have found abnormal brain information transmission of RBD and PD from the perspective of FC. However, there is a lack of consensus on the FC relationship between RBD and PD.

Recently, an analysis method of functional dynamics, measured by synchrony and metastability, has been used to analyze the efficiency and flexibility of brain information transmission [9]. Synchrony and metastability attempt to capture functional dynamics properties based on the temporal patterns of the oscillatory activity of brain regions [10, 11]. This is because synchrony in the oscillatory activity of network regions is thought to underpin information exchange [12], while metastability represents the variability in the synchronization of network regions over time that is considered essential for adaptive information processing [13, 14]. Currently, they have been applied to brain diseases, such as traumatic brain injury (TBI), Alzheimer's disease (AD), and schizophrenia [15–17]. Research has shown that PD's global synchrony and metastability are decreased compared with normal control (NC) [18]. However, the performance of resting-state network (RSN) functional dynamics in PD is unclear. Besides, the study of functional dynamics has only been reported in PD. There is a lack of research on RBD, including the difference in functional dynamics between RBD and PD.

In light of the foregoing, we used FC and functional dynamics to study the brain information transmission changes in RBD and PD patients. We performed FC and functional dynamics analysis in three aspects—based on the whole brain, within RSN, and the interaction between RSNs—using NC, RBD, and PD rs-fMRI data sets from Parkinson's Progression Markers Initiative (PPMI). Finally, a link between the neuroimaging measures and clinical features was sought across several clinical and neuropsychological assessments. Our study may help understand RBD and PD.

## 2. Materials and Methods

**Figure 1:**
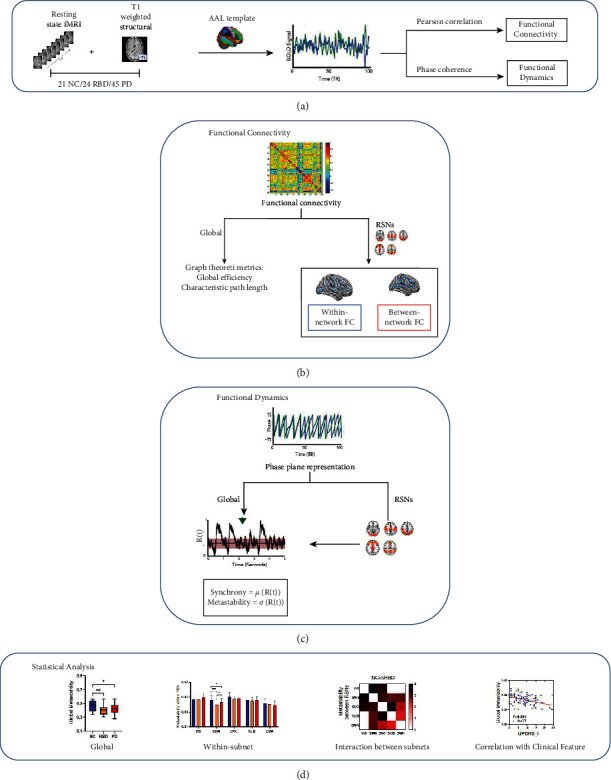
Technical route. (a) Functional image: data preprocessing and extraction time series. (b) Functional connectivity: calculation of global and RSNs indexes of FC. (c) Functional dynamics: calculation of synchrony and metastability from the perspective of global and RSNs. (d) Statistical analysis: analysis of neuroimaging measures differences and their relationship with clinical features.

The overall technical route is shown in Figure 1. Resting-state fMRI image was parcellated into 90 regions after preprocessing, from which the average BOLD signal was extracted. Then the BOLD signal was converted into FC through Pearson correlation, functional dynamics through phase coherence (see Figure 1(a)). The global FC was then characterized by graph-theoretic metrics. For RSNs, each RSN's within- and between-network FC was computed (see Figure 1(b)). We used the phase obtained from the signal to calculate the synchrony and metastability (see Figure 1(c)). Finally, we compared the differences in the global, within RSN and interactions between RSNs, and analyzed the relationship with clinical features (see Figure 1(d)).

### 2.1. Participants

In this study, the data used were selected from the publicly available Parkinson's Progression Markers Initiative (PPMI) dataset (https://www.ppmi-info.org) [19]. All subjects and their study partners completed the informed consent process. PD subjects were diagnosed according to the UK Parkinson's Disease Society Brain bank diagnostic criteria. All RBD cases were confirmed by polysomnography in line with the criteria proposed by the American Academy of Sleep Medicine. The presence of RBD was not assessed in the PD cohort. Subjects were stratified into three groups: 21 normal controls (NC), 24 rapid eye movement sleep behavior disorders (RBD) that were no Parkinson's symptoms, and 45 Parkinson's disease (PD). The detailed information is shown in Table 1.

### 2.2. Image Acquisition

These participants were enrolled at four PPMI sites that used a standardized protocol for three Tesla machines (all Siemens Healthcare, United States). Each resting-state session lasted about 8.4 min (210 volumes, TR = 2.4 s, TE = 25 ms, flip angle 80) with a voxel size of 3.3 × 3.3 × 3.3 mm (40 slices). Subjects were instructed to rest quietly, keep their eyes open, and not fall asleep. MPRAGE scans were also obtained (voxel size 1 × 1 × 1 mm, TR = 2.3 s, TE = 2.98 ms, flip angle = 9) for registration to the MNI template.

### 2.3. Image Preprocessing

Data preprocessing was carried out using Statistical Parametric Mapping (SPM12) and Data Processing Assistant for Rs-fMRI (DPARSF) toolkit [20]. The preprocessing steps were as follows: the first ten volumes of the functional images were discarded for fMRI signal equilibrium and the participants' adaptation to the scanning circumstance; the slice-timing correction was performed, the images were realigned for head movement; the effect of nuisance covariates was removed, including the global signal, the cerebrospinal fluid, and the white matter signals; the images were normalized to the Montreal Neurological Institute (MNI) space (resampled into 3 mm × 3 mm × 3 mm voxels); then the signal drift was removed. Finally, temporal band-pass filtering (0.01–0.21 Hz) was performed on the residual time series to reduce the effect of low-frequency drifts and high-frequency noise.

### 2.4. Cortical Parcellation

The automated anatomical labeling (AAL) Atlas [21] was used to define the regions of interest (ROIs). This Atlas contains 45 cortical and subcortical areas in each hemisphere (90 areas in total), alternatively interspersed (available by request). To acquire the total signal of a given ROI, it is necessary to compute an average over the entire time series of all the voxels of a given brain area following the AAL Atlas. In each subject, ROIs were assigned to the visual network (VIS), sensorimotor network (SMN), combining the regions of the dorsal and ventral attention network and control network (DVC), subcortical network (SUB), and default mode network (DMN).

### 2.5. Functional Connectivity

After the average time series of each ROI was extracted, the Pearson correlation between the ROIs in the whole brain was calculated to obtain functional connectivity (FC). The global topology of FC was evaluated using several graph-theoretic metrics. Calculation of graph-theoretic metrics was performed by using GRETNA [22]. Here, we selected the equal-interval sparsity threshold range (ranging from 0.1 to 0.5 with a partition interval of 0.05). We explored the following network topological attributes: global efficiency—the ability of a network to transmit information at the global level; characteristic path length—the extent of overall communication efficiency of a network [22].

Within-network FC for each RSN was computed by averaging the Pearson's correlation between the time series of all the voxels of the ROIs assigned to each particular network. For between-network FC, we first calculated an average time series within each RSN (as described above). We then computed the Pearson's correlation between the time series of each network and all the other networks [17].

### 2.6. Synchrony and Metastability

In our paper, functional dynamics were measured by synchrony and metastability. We used the Hilbert transform to convert the time series into a complex phase plane representation and then computed the order parameter *R*(*t*), defined as

(1) $$\begin{array}{c} R\left(t\right)=\left|\frac{1}{N}\sum_{n=1}^{N}{ei}^{\varphi_{n}\left(t\right)}\right|, \end{array}$$

where *N* is the total number of regions within the network and *φ*_*n*_(*t*) is the instantaneous phase of the regional mean BOLD time series at region *n*. The level of synchrony between phase time series is described by *R*(*t*) in terms of how coherently the phase changes over time [10, 23]. During fully synchronous behavior, *R*(*t*) = 1, whereas *R*(*t*) = 0, where the phase across all phase time series is fully asynchronous. We considered the mean of the order parameter *R*(*t*) across time as an index of synchrony and the standard deviation of the *R*(*t*) as an index of metastability.

Given that interconnected RSN works convey more behaviorally relevant information than single RSN works observed in isolation. We calculated local measures of network dynamics for the phase time series of regions defined in each of the resting-state brain networks as previously described: (1) the set of regions comprising single RSN and (2) when evaluating their interactions, the set of regions comprising two RSNs, where N refers to the number of brain regions included in the two RSNs [16].

### 2.7. Statistical Analysis

Statistical testing was performed by using the software SPSS. The differences in neuroimaging measures among the three groups were analyzed by using the nonparametric Kruskal–Wallis one-way analysis of variance. If significant results were reached by the variance analysis, multiple comparisons were made through the Mann–Whitney *U* test. The significance level was set at 0.05.

Accordingly, we applied the NBS to FC and functional dynamics evaluated at the between RSNs. Pairwise interactions between all five RSNs were evaluated using the approach described above. We applied the NBS to 21 5 × 5 symmetric matrices derived from NC and 24 5 × 5 symmetric matrices derived from RBD, and 45 5 × 5 symmetric matrices derived from PD. Each row/column represented an interaction between an RSN and four others of the matrix.

## 3. Results

### 3.1. Global Differences between Groups

**Figure 2:**
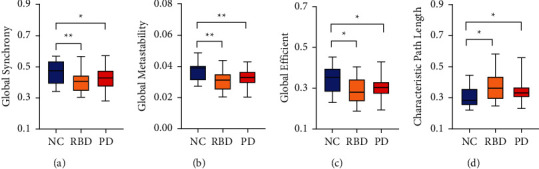
The differences in global neuroimaging measures between groups. (a) The difference in global synchrony between groups. (b) The difference in global metastability between groups. (c) The difference in global efficiency between groups. (d) The difference in characteristic path length between groups (^*∗*^*p* < 0.05, ^*∗∗*^*p* < 0.01).

Kruskal–Wallis one-way analysis identified a statistically significant difference between three groups in synchrony (*p*=0.019), metastablity (*p*=0.004), global efficiency (*p*=0.033), and characteristic path length (*p*=0.033). Subsequent Mann–Whitney test revealed, global synchrony (RBD: *p*=0.008, PD: *p*=0.026), metastability (RBD: *p*=0.003, PD: *p*=0.004), and efficiency (RBD: *p*=0.021, PD: *p*=0.033) was significantly lower in RBD and PD patients compared with NC (see Figures 2(a)–2(c)). Conversely, the characteristic path length (RBD: *p*=0.020, PD: *p*=0.034) was significantly higher in RBD and PD patients(see Figure 2(d)). All differences have been corrected.

### 3.2. Differences within RSN between Groups

**Figure 3:**
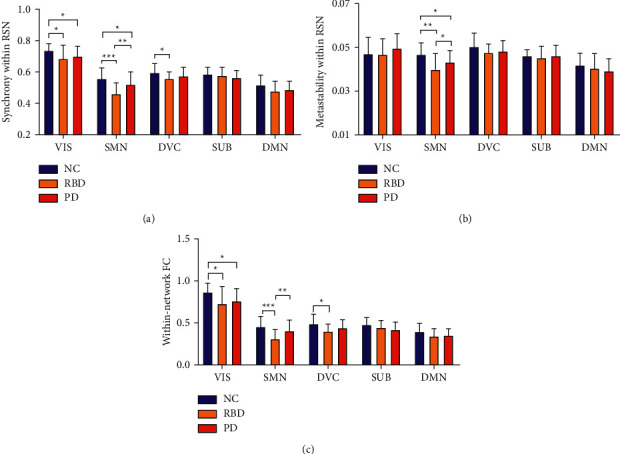
The differences in neuroimaging measures within RSNs between groups. (a) The difference in synchrony within RSN between groups. (b) The difference in metastability within RSN between groups. (c) The difference in within-network FC between groups. (^*∗*^*p* < 0.05, ^*∗∗*^*p* < 0.01, ^*∗∗∗*^*p* < 0.001).

At the RSNs level, RBD patients showed lower synchrony (VIS: *p*=0.019, SMN: *p*=0.000, DVC: *p*=0.017), lower metastability (SMN: *p*=0.001), and lower FC (VIS: *p*=0.011, SMN: *p*=0.000, DVC: *p*=0.012) in all five RSNs compared with NC (see Figure 3). PD patients showed lower synchrony (VIS: *p*=0.018, SMN: *p*=0.019), lower metastability (SMN: *p*=0.045), and lower FC (VIS: *p*=0.004) compared with NC (see Figure 3). Especially, RBD patients showed lower synchrony (*p*=0.001) and metastability (*p*=0.026) and FC (*p*=0.001) in SMN compared with PD (see Figure 3). All differences have been corrected.

### 3.3. Differences in Interaction RSNs between Groups

**Figure 4:**
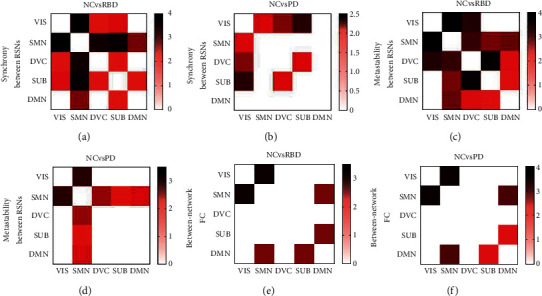
The differences in interaction RSNs between groups. (a) The difference in synchrony between RSNs (NC vs. RBD). (b) The difference in synchrony between RSNs (NC vs. PD). (c) The difference in metastability between RSN (NC vs. RBD). (d) The difference in metastability between RSNs (NC vs. PD). (e) The difference in between-network FC (NC vs. RBD). (f) The difference in between-network FC (NC and RBD); only significant columns are shown (*p* < 0.05, corrected); the value on the color bar represents the *t* value.

The NBS results showed significantly widespread decreases in synchrony (*p*=0.007; corrected) and metastability (*p*=0.003; corrected) that included the interaction between all RSNs in RBD compared with NC (see Figures 4(a) and 4(c)). Compared with NC, the decrease of PD in synchrony (*p*=0.021; corrected) is concentrated on VIS and other RSNs (see Figure 4(b)), in metastability (*p*=0.016; corrected) is focused on SMN and other RSNs (see Figure 4(d)). Compared with NC, the between-network FC decreased in RBD (*p*=0.041; corrected) and PD (*p*=0.025; corrected) patients. These inclusion decreases between SMN and VIS, SMN and DMN, DMN and SUB (see Figures 4(e) and 4(f)).

### 3.4. Correlations between Neuroimaging Measures and Clinical Features

**Figure 5:**
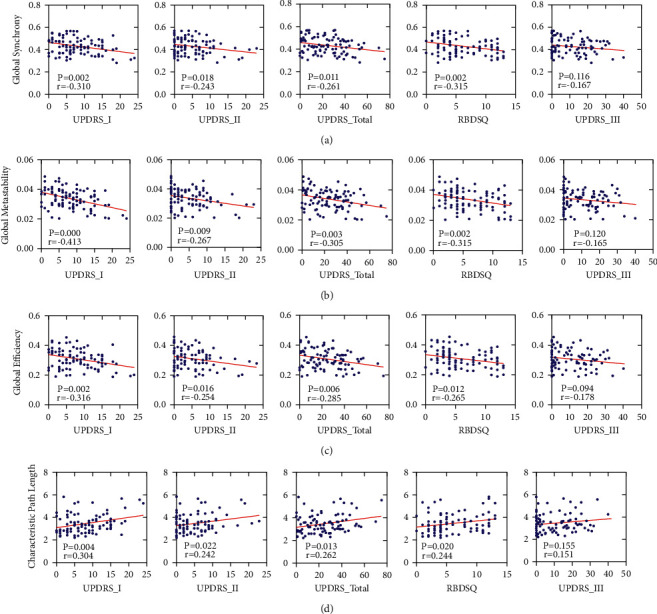
Correlations between neuroimaging measures and clinical features. (a) Correlation between global synchrony and UPDRS-I, II, III, total, RBDSQ. (b) Correlation between global metastability and UPDRS-I, II, III, total, RBDSQ. (c) Correlation between global efficiency and UPDRS-I, II, III, total, RBDSQ. (d) Correlation between characteristic path length and UPDRS-I, II, III, total, RBDSQ.

To investigate whether neuroimaging measures relate to the clinical features, we used clinical features (UPDRS-I, II, III, Total and RBDSQ and MoCA) as the dependent variable, global neuroimaging measures (synchrony, metastability, efficiency, and characteristic path length) as predictors, and age as covariates of no interest to carry out multiple linear regression. Global synchrony was negatively related to neuroimaging measures, including UPDRS-I (*r* = -0.310, *p*=0.002), UPDRS-II (*t* = −0.243, *p*=0.018), UPDRS-Total (*t* = −0.261, *p*=0.011) and RBDSQ (*t* = −0.315, *p*=0.002) (see Figure 5(a)). Global metastability was negatively related to neuroimaging measures, including UPDRS-I (*t* = −0.413, *p*=0.000), UPDRS-II (*t* = −0.267, *p*=0.009), UPDRS-Total (*t* = −0.305, *p*=0.003), and RBDSQ (*t* = −0.315, *p*=0.002) (see Figure 5(b)). Global efficiency was negatively related to neuroimaging measures, including UPDRS-I (*r* = −0.316, *p*=0.002), UPDRS-II (*t* = −0.254, *p*=0.016), UPDRS-Total (*t* = −0.285, *p*=0.006), and RBDSQ (*t* = −0.265, *p*=0.012) (see Figure 5(c)). Characteristic path length was positively related to neuroimaging measures, including UPDRS-I (*r* = 0.304, *p*=0.004), UPDRS-II (*t* = 0.242, *p*=0.022), UPDRS-Total (*t* = 0.262, *p*=0.013), and RBDSQ (*t* = 0.244, *p*=0.020) (see Figure 5(d)). There was no significant correlation between global neuroimaging measures and UPDRS-III, MoCA. To some extent, global neuroimaging measures can predict clinical features.

We also did the same for local neuroimaging measures. The results are shown in Table 2. The RSNs where neuroimaging measures (synchrony, metastability, within- and between-network FC) were decreased, including VIS and SMN and DVC, were negatively associated with the clinical features, including UPDRS-I, II, Total, and RBDSQ. In particular, DMN synchrony (*r* = −0.215, *p*=0.037) and FC (*r* = −0.218, *p*=0.039) were negatively correlated with UPDRS-I. DMN metastability was negatively correlated with UPDRS-I (*r* = −0.235, *p*=0.023), UPDRS-II (*r* = −0.251, *p*=0.015), and UPDRS-Total (*r* = −0.246, *p*=0.017).

## 4. Discussion

This paper studied brain information transmission changes in patients with RBD and PD by two methods—FC and functional dynamics. Compared with NC, the global functional dynamic and efficiency of RBD and PD were decreased and the characteristic path length was increased (see Figure 2). The local decrease was concentrated in SMN and VIS, while RBD had more extensive damage to SMN than PD (see Figure 3). On the functional dynamics between RSNs, RBD showed a widespread decrease, and PD showed a focal decrease which concentrated in SMN and VIS (see Figure 4). The between-network FC decreased in RBD and PD, including decreases between SMN and VIS, SMN and DMN, DMN and SUB (see Figure 4). Finally, we found that the neuroimaging measures used in our study were linked to motor and nonmotor symptoms in patients (see Figure 5, Table 2).

### 4.1. Global Information Transmission Efficiency and Flexibility Are Decreased in RBD and PD

We used FC and functional dynamics to study the changes in brain information transmission. FC is the temporal coherence of neuronal activity patterns emerging from anatomically separated brain regions. Global efficiency and characteristic path length of the FC network reflect the extent of global information transmission efficiency of the brain. Because the brain is a complex dynamical system [13, 24], communication between neural ensembles is achieved through coherence, whereby two neural assemblies whose activity fluctuates in synchrony can exchange information [12]. Therefore, synchrony relates to the information interaction efficiency of the brain. Metastability refers to the variability of synchrony over time. Thus, it reflects the flexibility of brain information transmission [9].

In our study, compared with NC, the global efficiency and functional dynamics of RBD and PD were decreased, and the characteristic path was increased (see Figure 2). These indicate that the patient's brain information transmission efficiency is reduced. This notion is supported by studies that found that whole brain activity in RBD and PD is characterized by a less efficient state [18, 25, 26]. Low metastability suggests that information transmission flexibility is low, and the activity in patients is more rigid and less variable. The evidence suggested that PD and RBD stayed longer in a weakly connected state and tended to have a decreased number of transitions, indicating a sparsely connected brain network with a relative loss of brain dynamics [27, 28]. The decrease of metastability was found in the study of mental diseases, which was related to the impairment of cognitive ability [15, 16]. However, the relationship between metastability and behavior was not specific to cognitive ability [15]. In our study, we demonstrated that global synchrony and metastability negatively correlated with the clinical features of PD, which include UPDRS-I, II, Total, and RBDSQ (see Figure 5). These activities require communication between sensory, motor, and cognitive control regions, so the decline of information transmission efficiency and flexibility indicates the deterioration of clinical manifestations. The above results suggest that global synchrony and metastability may be an essential dynamical mechanism underlying general motor and nonmotor symptoms in RBD and PD.

Interestingly, we found that RBD had a greater downward trend than PD in global FC and functional dynamics (see Figure 2). The possible reason is that the functional change that evolves over months to years from RBD to PD is nonlinear. For example, in PD, it is known that there is a complex relationship between the specific cognitive problems faced by patients and the specific stage of their disease [29]. There may be a compensatory mechanism.

### 4.2. RBD Has More Extensive Damage to SMN than PD

To our knowledge, this is the first study on RSN synchrony and metastability in RBD and PD, and the results reinforce the importance of SMN for RBD and PD patients. The SMN comprises the primary sensorimotor cortex as well as areas involved in motor task preparation, such as the premotor cortex and the SMA, and is activated in tasks of voluntary movements [7]. In our study, the synchrony, metastability, and FC of SMN were lower in patients (see Figure 3). It reflects the impaired brain information transmission of SMN in patients. Neuroimaging studies have repeatedly shown disease-related alterations in sensorimotor areas in RBD and PD [7, 8, 30]. In particular, we found that the synchrony and metastability and FC in SMN of RBD were lower than those of PD. It may imply the compensatory mechanism, which has been found in the literature. For example, some papers proved that with the development of PD, partial connections of motor brain regions are strengthened, suggesting ongoing attempts of recovery and compensatory mechanism for affected functions [31]. In the interactive network, the functional dynamics between SMN and other RSNs were decreased in RBD and PD (see Figures 4(a)–4(d)). They highlighted that disruption of sensorimotor integrative function is driven not only by changes within the network but also by large-scale network-to-network disconnections [7]. Not only the internal information transmission of SMN is restricted, but also the information transmission between SMN and other RSNs are reduced in patients, which weakened the interaction between the subnetwork of brain and affected the overall function of the brain.

We did not observe pronounced correlations of UPDRS-III with functional indexes within SMN might indicate that FC and functional dynamics of the SMN could be present independently from motor symptom severity in RBD and PD. On the other hand, it is conceivable that diverging pathological effects exist throughout different stages of the disease and across different motor subtypes, which lead to similar motor severity but different FC and functional dynamics alterations of the SMN [7]. It is worth noting that the UPDRS-III score of PD was significantly higher than that of RBD, while the functional connection and functional dynamics of the SMN that of RBD were significantly lower than that of PD. These results corroborate the independence of the motor-associated property. In this case, more pronounced correlation effects could be obscured in our study's rather heterogeneous patient sample.

### 4.3. The Decline of Functional Dynamics in VIS and DVC Is Accompanied by the Decline of FC

Our results showed that the synchrony within RSN was decreased in VIS and DVC (Figures 3(a) and 3(b)). This can also be observed in the results of FC (see Figure 3(c)). PD is associated with a broad range of visual symptoms [32], such as RBD, visuospatial disorders, and visual hallucinations. RBD patients are always accompanied by nightmares, which may be related to visual hallucinations during dreaming [33] With the recognition of RBD, increasing evidence has suggested that visually related areas are closely associated with the pathophysiology of RBD, and further so in motor control [34]. The DVC includes dorsal and ventral attention and control networks. Similar to our study, Zhang also reported that patients with RBD have a functional decline in attention, executive function, contextual verbal memory, and nonverbal memory [3]. Brain connection pathways of patients are reduced, and brain information transmission is restricted in VIS and DVC. Equally, we found that the neuroimaging measures in VIS and DVC were related to part clinical features (see Table 2). Compared with NC, the interaction between VIS and other RSNs was damaged in RBD and PD. These damages were consistent with the results of within RSN. It shows that the RSN interaction damage in the interactive network may also be related to the RSN internal function damage [7, 35]. Damage within VIS can result in reduced information transmission with other RSNs.

### 4.4. The Neuroimaging Measures in DMN Predict Nonmotor Symptoms in RBD and PD

The DMN is characterized by basal activity that increases during rest or passive visual fixation and decreases (“deactivates”) during cognitive tasks [36]. It appears to be particularly vulnerable to the effects of the disease and has been reported on PD before [37]. But, in the current study, RBD and PD exhibited no abnormalities in DMN. Unfortunately, there are no clear patterns regarding the default mode network in RBD and PD are present in the literature [30, 38]. Some papers report no alterations of DMN [30, 37]. However, a study aiming to distinguish PD patients from healthy subjects found rather substantial alterations of the DMN in disease [39]. These conflicting findings could be ascribed to differences in stage, clinical phenotype, and treatment type in the different PD cohorts studied [40]. In addition, we found that neuroimaging measures in DMN were significantly negatively correlated with UPDRS-I, that is, the score of the mood and cognition-related scale (see Table 2). Therefore, the neuroimaging measures in DMN can indeed reflect the severity of nonmotor symptoms in patients [38].

## 5. Conclusions

In summary, we studied brain information transmission of RBD and PD by two methods—FC and functional dynamics. We found differences in synchrony, metastability, and FC between NC and patients. These differences prove that brain information transmission efficiency and flexibility are impaired in RBD and PD. This impairment is not only found in functional dynamics but also implied to be related to behavior.

## Figures and Tables

**Table 1:** Demographic and clinical characteristics of all subjects.

	NC (*n* = 21)	RBD (*n* = 24)	PD (*n* = 45)	*p*-value
Gender (M : F)	17 : 4	21 : 3	24 : 21	0.003
Age (years)	65.0 ± 9.3	71.6 ± 5.5	60.3 ± 7.4	0.000
UPDRS_I	2.6 ± 2.0	11.7 ± 5.0	9.1 ± 5.0	0.000
UPDRS-II	0.7 ± 1.8	4.6 ± 5.1	7.2 ± 4.9	0.000
UPDRS_III	1.0 ± 1.5	4.4 ± 5.7	19.0 ± 7.9	0.000
UPDRS_total	4.3 ± 4.1	20.6 ± 12.4	35.3 ± 13.7	0.000
RBDSQ	3.2 ± 1.7	10.3 ± 2.2	5.0 ± 3.0	0.000
MoCA	28.0 ± 1.2	26.8 ± 2.4	27.0 ± 2.8	0.275

Abbreviations: NC : normal control; RBD : rapid eye movement sleep behavior disorder; PD : Parkinson's disease; UPDRS : new version of the world movement disorder society Parkinson's disease comprehensive rating scale (part I: mood/cognition, part II : activities of daily living, part III : motor examination, total : sum of parts I–III); RBDSQ : RBD symptom questionnaire; MoCA : montreal cognitive assessment.

**Table 2:** Correlations between neuroimaging measures within RSN and clinical features.

		UPDRS-I	UPDRS-II	UPDR-III	UPDRS-total	RBDSQ	MoCA
Synchrony	VIS	**−0.309 (0.002)**	**−0.250 (0.015)**	−0.172 (NS)	**−0.279 (0.007)**	**−0.355 (0.000)**	−0.041 (NS)
	SMN	**−0.342 (0.001)**	**−0.244 (0.018)**	−0.061 (NS)	**−0.221 (0.033)**	**−0.482 (0.000)**	0.038 (NS)
	DVC	−0.194 (NS)	−0.081 (NS)	0.038 (NS)	−0.064 (NS)	**−0.208 (0.045)**	−0.036 (NS)
	SUB	−0.029 (NS)	−0.112 (NS)	0.178 (NS)	−0.173 (NS)	0.038 (NS)	0.042 (NS)
	DMN	**−0.215 (0.037)**	−0.188 (NS)	−0.059 (NS)	−0.162 (NS)	−0.173 (NS)	−0.016 (NS)

Metastability	VIS	0.135 (NS)	0.134 (NS)	0.120 (NS)	0.157 (NS)	**0.236 (0.022)**	0.070 (NS)
	SMN	**−0.247 (0.016)**	−0.180 (NS)	−0.118 (NS)	**−0.205 (0.047)**	**−0.368 (0.000)**	−0.035 (NS)
	DVC	−0.156 (NS)	0.004 (NS)	−0.079 (NS)	−0.097 (NS)	−0.076 (NS)	0.032 (NS)
	SUB	0.091 (NS)	0.019 (NS)	−0.046 (NS)	0.007 (NS)	−0.053 (NS)	0.098 (NS)
	DMN	**−0.235 (0.023)**	**−0.251 (0.015)**	−0.156 (NS)	**−0.246 (0.017)**	−0.068 (NS)	0.021 (NS)

FC	VIS	**−0.306 (0.003)**	**−0.273 (0.009)**	−0.178 (NS)	**−0.317 (0.002)**	**−0.351 (0.001)**	−0.022 (NS)
	SMN	**−0.325 (0.002)**	**−0.259 (0.014)**	−0.088 (NS)	**−0.236 (0.025)**	**−0.459 (0.000)**	0.074 (NS)
	DVC	**−0.228 (0.031)**	−0.094 (NS)	0.013 (NS)	−0.094 (NS)	−0.193 (NS)	−0.018 (NS)
	SUB	−0.136 (NS)	−0.156 (NS)	0.190 (NS)	−0.168 (NS)	−0.045 (NS)	0.027 (NS)
	DMN	**−0.218 (0.039)**	−0.205 (NS)	−0.097 (NS)	−0.190 (NS)	−0.150 (NS)	0.007 (NS)

Significant correlations (*p* < 0.05) are highlighted in bold and marked with *p*-values; the remaining correlations are marked as not significant (NS).

## Data Availability

Data used in the preparation of this paper were obtained from Parkinson's Progression Markers Initiative (PPMI) database (https://www.ppmi-info.org/data). As such, the investigators within PPMI contributed to the design and implementation of PPMI and/or provided data but did not participate in the analysis or writing of this report.

## Additional Points

*Limitation*. This study is cross-sectional and retrospective, and we do not know if and when any of the RBD subjects will develop PD or another alpha-synucleinopathy. Thus, our results have to be interpreted with caution, and longitudinal studies involving larger samples are warranted to confirm our findings.
